# Prosecution for Manslaughter by Mal-Practice

**Published:** 1847-12

**Authors:** 


*Prosecution for Manslaughter by Mal-practice.*—A prosecution for manslaughter, in the fourth degree, in consequence of mal-practice, the defendant being a non-medical man, and professing in this instance only to be a practitioner, was tried before Judge Stevens, in this city, at the Erie County Court, a brief account of which may possess some interest for our readers. We condense the following from the minutes of Judge Stevens, which he has kindly placed at our disposal for that purpose:

It appears from the testimony, that in the latter part of August last, a man by the name of Edwin Backus, arrived at Hamburg, Abbot's Corners, near this city, in the public conveyance, being on his way to Jamestown, and stopped at the tavern, being too unwell to proceed farther. He stated that he had not been well for several days. The defendant, named George B. Green, residing at Abbott's Corners, was loitering at the tavern when he arrived, and, professing to be a practitioner of medicine, volunteered to prescribe for him, to which the man assented. Green then went to the office of Dr. Nott, which was nigh at hand, and requested of a medical student in the office, (Dr. Nott being absent,) to supply him with some blue pills, saying that he desired them for a friend of his who was ill at the tavern. While the student was looking for the pills, Green examined a vial containing oil of hemlock, which happened to be there, and after tasting it, and calling it oil of turpentine, said it was just what he wanted, and that he would take it instead of the pills. The vial was almost one-third full, (an ounce vial;) but, before leaving the office, Green added about two-parts of water. This vial he gave to the sick man, with directions to take a tea-spoonful every two or three hours, and received half a dollar for the advice and medicine. The patient then retired to bed. This was in the afternoon. In the evening he complained of great sickness, and the next morning, at daylight, of pain in his back, and in the region of the stomach, nausea, and sense of soreness in the stomach. Dr. Nott was sent for at 8 A. M. The vial was then examined, and about two-thirds of its contents found to have been taken. Dr. Nott ascertained that during the night he had suffered much from what he called severe cramps in the stomach, together with acute pains in the head, and back. The spasms occurred at intervals. There had also been epistaxis. He complained at the time of his visit, of nausea, and pain in the back. There was obscure tenderness over the epigastrium, and in right hypochondrium. The spasms and acute cephalalgia did not return. Dr. Nott administered an emetic, which appeared to afford relief.

Dr. Nott considered the case to be one of bilious fever. Remissions occurred several times, giving hope of recovery, which proved delusive, and death took place about four weeks after his arrival. During the latter part of his illness he vomited almost incessantly a bluish tinged fluid, which stained linen like blue ink. This continued until he died. The alvine discharges, at that time, were dark, like tar.

On examination after death, the stomach contained some of the same fluid vomited. The mucus coat throughout was softened, resembling jelly, and could be scraped off readily with the scalpel. Similar disorganization was observed in the duodenum, and upper portion of small intestine. The intestinal glands did not present disease. Liver and spleen were enlarged.

Dr. Pringle, of Hamburg, who attended the case in conjunction with Dr. Nott, concurred in the foregoing statement of symptoms, and morbid appearances.

Drs. Pringle and Nott testified, that in their opinions, the oil of hemlock aggravated the symptoms, and augmented the disease, but that it would be difficult to assign the precise amount of its agency in the fatal result.

Dr. A. S. Sprague, of this city, testified, that if the quantity taken had been pure oil, it might have produced death ; that a prescription of it in any quantity, in bilious fever, would be bad practice ; that to prescribe the same quantity of oil of turpentine would display gross ignorance.

Dr. C. Winne testified to the impropriety of the prescription ; that the article is poisonous, producing inflammation, convulsions, and stupor. It would have no other effect than to aggravate the disease.

Dr. M. Bristol testified that the practice of the defendant was bad ; that violent spasms, and inflammation of the stomach were to be expected as consequences, and that the chances of recovery were lessened by the administration of the remedy.

The defendant was convicted of *misdemeanor*, and sentenced by the court to confinement for three months in the county jail.

The object of the defendant, in prescribing for the patient, was, manifestly, the paltry fee which he succeeded in obtaining. Tampering with life, and for such a motive, our readers cannot but concur with us in saying should be severely punished. On the same principle how can the public overlook the dispensing of secret remedies by those wholly unacquainted with the laws of life and disease, and the therapeutic operation of remedies, for similar mercenary ends? We challenge any one to show any essential difference between the outrage of Green, in the instance just detailed, and that daily and hourly perpetrated by the vender of patent medicines, except that the latter deals destruction by wholesale. Yet, the public require that the one should be punished, and permit the other to go unscathed. Surely, the law abounds in "glorious" *inconsistencies*, as well as "uncertainties."

